# Association Between Maternal Body Mass and Physical Activity Counseling During Pregnancy

**DOI:** 10.3389/fpsyg.2021.612420

**Published:** 2021-11-25

**Authors:** Shelly Ruart, Stéphane Sinnapah, Olivier Hue, Eustase Janky, Sophie Antoine-Jonville

**Affiliations:** ^1^Laboratoire Adaptations au Climat Tropical Exercices et Santé (ACTES) (EA 3596), Université des Antilles, Pointe-à-Pitre, France; ^2^Service de Gynécologie Obstétrique, Centre Hospitalier Universitaire de la Guadeloupe, Pointe-à-Pitre, France

**Keywords:** weight problem, physical activity, gestational weight gain, counseling, prenatal care

## Abstract

**Aims:** The antenatal period provides an important opportunity for giving advice on healthy lifestyle choices. However, the prevalence of maternal obesity is increasing, and women report that they do not receive counseling. We investigated the information given to pregnant women on gestational weight gain, physical activity, and nutrition during pregnancy in relation with their initial weight status, current gestational weight gain and diagnoses of either pre-pregnancy overweight/obesity or excessive gestational weight gain.

**Methods:** Cross-sectional survey using a questionnaire. Pregnant participants (*n* = 141) were recruited from a midwife center. They completed a structured questionnaire on the information they received during their pregnancy and we assessed its relationship with their weight.

**Results:** We found that many pregnant women did not receive advice about physical activity, gestational weight gain and nutrition (37.5, 53.2, and 66.2%, respectively). Women with weight problems (pre-pregnancy overweight/obesity and excessive gestational weight gain) were less targeted for counseling, although more than 80% of the women viewed receiving information on these topics as positive. Also, being informed of a weight problem was associated with a greater chance of receiving information about physical activity, gestational weight gain and nutrition (all *p* < 0.05). However, verbalization of the weight problems was low (14.0% of women with pre-pregnancy overweight were informed of their status).

**Conclusion:** Health professionals should dispense more information, especially on PA and particularly for women with weight problems. Verbalization of the weight problem seems associated with more frequent transmission of information.

## Introduction

Physical activity (PA) prevents excessive gestational weight gain (excessive GWG) (Ruchat et al., 2018) and many of the complications of pregnancy (Hinman et al., 2015), and it is recommended throughout pregnancy (ACOG Committee Opinion No. 650, 2015). However, the quantity is usually insufficient and tends to decline with the trimesters (Coll et al., 2016). Many determinants of this decline have been documented, notably a lack of advice and information on the effects of PA during pregnancy (Coll et al., 2017; Thompson et al., 2017). Health professionals find that most of these determinants are hard to modify within the healthcare framework, but the lack of information, which women have cited to explain their low PA in several studies (Connelly et al., 2015), seems modifiable. More information might thus be a potential public health target.

The pregnancy weight risk may be due to pre-pregnancy overweight (Samura et al., 2016) and/or excessive weight gain during pregnancy (Ren et al., 2018). However, several studies have reported that clinicians do not systematically diagnose or discuss overweight (Duthie et al., 2013; Nikolopoulos et al., 2017). The clinical recommendations for managing obese patients in general include obesity screening and assessment in primary care practice, and health professionals are encouraged to identify those patients who need to lose weight (Kushner and Ryan, 2014). Excessive GWG has been well-documented (Deputy et al., 2015) and is indeed a priority issue, as the recommendation is to control weight gain throughout pregnancy (Institute of Medicine (US) and National Research Council (US) Committee to Reexamine Iom Pregnancy Weight Guidelines, 2009). Yet, according to women’s reports, discussions on GWG occur in less than 50% of pregnancies (McDonald et al., 2011; Deputy et al., 2018). Furthermore, few pregnant women reported receiving advice in according recommendation or discussed the risks of inappropriate GWG with health professionals (Morris et al., 2017; Whitaker et al., 2020). The lack of counseling is not without consequence because women who did not report advice by their health professionals on GWG were at higher risk for both inadequate and excessive GWG (Whitaker et al., 2020). Similar to that of GWG−related counseling, the quantity of PA information is insufficient and often inappropriate (Stengel et al., 2012; Lindqvist et al., 2018).

The objective of this study was to investigate the information given to pregnant women on gestational weight gain, physical activity, and nutrition during pregnancy in relation with their initial weight status, current GWG and diagnoses of either pre-pregnancy overweight/obesity or excessive GWG. We hypothesized that weight problems would increase the frequency of counseling for pregnant women. Secondly, we investigated pregnant women’s beliefs and attitudes toward PA with the objective of improving the targeting and content of prenatal PA counseling.

## Materials and Methods

### Study Design and Participants

This was a cross-sectional study design, with 141 pregnant women from a midwife center in Guadeloupe. After giving informed consent, the women responded to a structured questionnaire under their midwife’s supervision on the counseling they had received and their beliefs about PA during pregnancy. Inclusion criteria were being pregnant; able to read, write and speak French; followed throughout pregnancy in the center where the recruitment took place; and with no contraindication to PA according to the ACOG. All experimental procedures conformed with the Declaration of Helsinki and were approved by the local ethics committee.

### Ethical Approval

The study was conducted in accordance with the Declaration of Helsinki and the current local regulations. All participants provided written informed consent. It was emphasized that participation was voluntary and that they could withdraw from the project at any time with no explanation required. No financial support was given.

### Data Collection

On the day of data collection, all eligible and available women were invited to participate in the study. The progress of pregnancy in weeks of amenorrhea and trimesters of pregnancy, stature in centimeters, current weight status, age in years, GWG in kilograms at the time of the study, and weight in kilograms as beginning pregnancy were collected from medical records. The women were also interviewed during visits to the midwife center using a questionnaire built for the current study. The information from the participants about PA, nutrition, and GWG was examined. We developed the questionnaire based on questions and results from similar studies (Lindsay et al., 2017; Vinturache et al., 2017). Although the questionnaire did not first undergo a full validation procedure, all question-and-answer options were piloted in 20 pregnant women at various stages of pregnancy for comprehensibility. The content of the questionnaire was revised base on the feedback on the women to improve the readability and understanding. Some questions were duplicated to check for understanding. Some questions were not used for the final questionnaire. In the initial version, open-ended questions were used to optimize the phrasing of the questionnaire.

The questionnaire was composed of a mix of closed-ended questions, semi-closed-ended questions, and 5-point Likert scale questions. It required approximately 10 min to complete.

The frequency of advice received (on nutrition, GWG, and PA) was examined on participants on a scale of 0–5, where 0 represented “never” and 5 “very frequently.” Did you receive advice during your consultations on nutrition, GWG and PA?

To assess whether women had received information about their weight problems, closed questions were used. “Were you informed about an overweight/obesity problem before pregnancy by your midwife?”, “Were you informed about excessive GWG during pregnancy by your midwife?”

#### Assessment of Pre-pregnancy Body Mass Index and Excessive Gestational Weight Gain

Pre-pregnancy BMI was calculated using pre-pregnancy weight and height, and the women were categorized as underweight (BMI: <18.5 kg/m^2^), normal weight (BMI: 18.5–24.9 kg/m^2^), overweight (BMI: 25.0–29.9 kg/m^2^) or obese (BMI: ≥30.0 kg/m^2^). The total GWG was calculated from the maternal weight. Based on the ACOG recommendations (ACOG Committee opinion no. 548, 2013), total GWG was defined as excessive if above the upper limit determined for each BMI class as 18.0, 16.0, 11.5 and 9.0 kg in pre-pregnancy underweight, normal weight, overweight and obese mothers, respectively. The weight gain was subsequently considered not excessive if within the ACOG recommendations (12.5–18, 11.5–16.0, 7.0–11.5, and 5.0–9.0 kg, respectively).

### Statistical Analysis

The demographic characteristics were analyzed as means and standard deviations (SD) for quantitative variables and confidence intervals for qualitative variables. Average and frequency comparisons were used for qualitative and quantitative variables, respectively. Variables evaluated by Likert scales were recoded into dichotomous variables (“expresses an agreement” and “does not express an agreement”) because of the small sample size. Odds ratios were calculated when Chi^2^ analyses revealed significant associations. The effect of pre-pregnancy overweight/obesity diagnosis or excessive GWG on the frequency of information received (on weight gain, nutrition, and PA) was explored by Chi^2^ tests. All questionnaire data were entered into Excel software and imported into SPSS (v24.0). The results were considered significant when the *p*-value was ≤ 0.05.

## Results

### Participants

One hundred and forty-one 30.3 ± 6.2-year-old women between 10 and 42 weeks of gestation participated in the present study. They were predominantly married (61.0%) and employed (63.1%). The majority had completed secondary school (70.2%) and 51.1% had at least one child. Before pregnancy, 9.3% (95%CI 4.5–14.1), 50.0% (95%CI 41.7–58.3), 26.4% (95%CI 19.1–33.7) and 14.3% (95%CI 8.5–20.1) were lean, normal weight, overweight and obese, respectively. The expected maximum weight gain throughout the pregnancy was 8.2 ± 7.7 kg and by the time of the study, 13.5% (95%CI 7.8–19.2) showed excessive GWG. Nevertheless, 77.3% (95%CI 70.4–84.2) considered themselves in good health. The distribution of BMI into classes before pregnancy was as follows: 9.3% (95%CI 4.5–14.1), 50.0% (95%CI 41.7–58.3), 26.4% (95%CI 19.1–33.7), and 14.3% (95%CI 8.5–20.1) in lean, normal, overweight and obese women, respectively. A significant relationship between being pre-pregnancy overweight or obese and excessive GWG was evidenced (*p* < 0.05, Table 1).

**TABLE 1:** Excessive gestational weight gain (GWG) for body mass index (BMI) respondents ≥25 and ≥30, respectively, before pregnancy, vs. respondents with BMI <25.

	**BMI ≥ 25 pre-pregnancy OR (95%CI)**	**BMI ≥ 30 pre-pregnancy OR (95%CI)**
Excessive GWG	2.89 (1.06–7.88)	3.39 (1.09–10.49)

### The Relationship of the Women With the Information

The women most frequently reported that the main source of information was press/books/internet [51.8% (95%CI 43.6–60.0)], followed by family and friends [28.4% (95%CI 21.0–35.8)] and last by the medical professions [19.8% (95%CI 13.1–26.4)].

Most of the women [88.6% (95%CI 83.4–93.8)] thought PA was an important topic and 83.2% (95%CI 77.0–89.4) and 84.4% (95%CI, 78.4–90.4), respectively, thought they had enough time and felt comfortable enough to ask questions about PA in the consultations. However, 74.5% (95%CI 67.3–81.7) would have liked to know more. Regarding the advice given, 25.5% (95%CI 18.2–32.7) and 31.2% (95%CI 23.6–38.8) reported difficulties in following the advice on PA and nutrition, respectively.

Gestational weight gain was considered an important topic by 65.2% (95%CI 57.3–73.1) of the women. Most [87.2% (95%CI 81.7–92.7)] thought receiving information on their weight was positive, and 56.1% (95%CI 47.9–64.3) reported trying to control their weight gain.

### Information Received by the Women

In sample, 53.2% (95%CI 45.0–61.4) reported receiving information on GWG, 66.2% (95%CI 58.4–74.0) on nutrition, and 37.5% (95%CI 29.5–45.5) on PA from their health professionals. Respectively, 67.2% (95%CI 59.5–74.9) and 65.0% (95%CI 57.1–72.9) of the women said they were aware of the impact of PA and nutrition on GWG. With regard to weight gain, 26.7% (95%CI 19.4–34.0) were informed of a weight gain not to be exceeded. When asked about the maximal weight gain that they had been advised not to overpass, 88.7% (95%CI 83.4–93.9) reported a gain that was in the range of the IOM recommendations. Among the women who received PA information, 89.4% (95%CI 84.3–94.5) reported that their doctor spoke positively about PA. PA was assumed to be positive for the mother and the fetus by, respectively, 90.1% (95%CI 85.2–95.0) and 80.7% (95%CI 74.2–87.2). The frequency of reporting the PA benefits for their baby’s health was higher in women who had received information about PA, whereas the frequency of reporting PA as dangerous was lower (Table 2). Results not displayed were higher than *p* ≥ 0.323.

**TABLE 2:** Distribution of information on physical activity and gestational weight gain.

	**Received GWG information**			**Received PA information**		
	**Yes (%)**	**No (%)**	**OR (95%CI)**	**Yes (%)**	**No (%)**	**OR (95%CI)**
At ease asking the midwives questions	91.9	77.3	3.33 (1.20–9.18)^*^	92.0	80.0	2.87 (0.90–9.05)^*^
Received information about her nutrition	93.2	36.4	23.80 (8.43–67.16)^**^	96.1	47.1	27.56 (6.39–120.67)^**^
Informed of excessive GWG	20.0	6.1	3.87 (1.21–12.34)^*^	29.4	3.5	11.38 (3.10–41.79)^**^
Received PA information	60.3	11.1	12.13 (4.86–30.30)^**^			
Received GWG information				86.3	34.1	12.13 (4.86–30.30)^**^
Declared that PA is good for baby’s health				88.2	75.0	2.50 (0.93–6.69)^*^
Declared less frequently that PA is dangerous				21.6	37.6	1.91 (0.82–4.43)^*^

*p < 0.05; **p < 0.01.

### Which Women Received Information About Weight?

Fourteen percent (95%CI 8.3–19.8) of the women who were overweight or obese before pregnancy had been informed of their status, and 31.6% (95%CI 28.1–44.0) of the women who had gained too much pregnancy weight by the time of the study had been informed about it. Not all the women received information about GWG but giving information tended to increase with pregnancy progression: 27.8% (95%CI 20.4–35.2), 48.3% (95%CI 40.0–56.6) and 65.1% (95%CI 57.2–73.0) for the first, second, and third trimesters, respectively (*p* = 0.005).

Women who felt at ease about asking questions (see Table 2) and women who were informed that they had gained too much weight during pregnancy were more likely to receive information about GWG (see Table 3). Women with pre-pregnancy overweight or excessive GWG did not receive information about GWG more frequently than the other women (see Table 3).

**TABLE 3:** Likelihood of receiving information on gestational weight gain, physical activity, and nutrition according to the pre-pregnancy BMI (normal or not), gestational weight gain (GWG; excessive or not), and information on excessive GWG (received or not).

		**Pre-pregnancy BMI ≥ 25**			**Informed of BMI ≥ 25**			**Excessive GWG**			**Informed excessive GWG**		
**Received information**		**Yes (%)**	**No (%)**	**OR (95% CI)**	**Yes (%)**	**No (%)**	**OR (95% CI)**	**Yes (%)**	**No (%)**	**OR (95% CI)**	**Yes (%)**	**No (%)**	**OR (95% CI)**
	GWG	52.6	54.2	0.93 (0.47–1.84)	34.8	39.7	0.81 (0.40–1.64)	57.9	52.5	1.26 (0.46–3.31)	78.9	49.2	3.87 (1.21–12.34)^*^
	PA	34.5	40.0	0.72 (0.38–1.61)	33.3	39.2	0.77 (0.36–1.64)	29.4	38.7	0.66 (0.21–2.0)	83.3	30.5	11.38 (3.10–41.79)^*^
	Nutrition	64.9	67.9	0.87 (0.42–1.79)	34.5	43.5	0.68 (0.32–1.43)	77.8	64.5	1.92 (0.59–6.22)	79.3	20.7	0.60 (0.52–0.70)^*^

*p < 0.05.

### Which Women Received Information About Physical Activity?

Information about PA was more frequently received as the pregnancy progressed, with 16.7% (95%CI 10.5–22.9), 32.8% (95%CI 25.0–40.5) and 48.3% (95%CI 40.0–56.5) of the women being informed for the first, second and third trimesters, respectively: *p* = 0.016.

The analyses failed to evidence any tendency of PA information to be more frequently received by women with pre-pregnancy overweight or excessive GWG (Table 3). However, women who were informed that they had gained too much weight during pregnancy were more likely to receive information on PA and nutrition (Table 3).

## Discussion

The objective of this study was to investigate the information that pregnant women receive with regard to their initial weight status, pregnancy weight gain and weight diagnosis. We hypothesized that weight problems would increase the frequency of counseling for these women. The results highlight that many pregnant women did not receive advice about PA, GWG and nutrition. In addition, women who had a pre-pregnancy weight issue were less targeted for counseling.

### A Little Discussed Subject

The proportion of women who declared that they had received information on gestational weight gain or physical activity by a medical professional was quite low, even though it increased as the pregnancies progressed. In the third trimester, only 65.1 and 48.3% of the women said they had heard of gestational weight gain and physical activity, respectively. These frequencies may be surprising, given the importance of these two parameters over the course of a normally progressing pregnancy and childbirth, but they approximately reflect the frequencies found in the literature, with values ranging from 21 to 52% for gestational weight gain (McDonald et al., 2011; Whitaker et al., 2016b; Morris et al., 2017) and from 50 to 63% for physical activity (de Jersey et al., 2013; Whitaker et al., 2016b). This may have had direct consequences on the women’s physical activity practice during pregnancy as some studies have shown that the desire to exercise during pregnancy is higher among women followed by obstetricians who discuss PA during prenatal visits (May et al., 2013; Nascimento et al., 2015). Potential explanations for the insufficient prenatal counseling have been much explored and discussed in the literature. Is this subject too delicate, uninteresting/useless, or too complex?

### A Delicate Subject

One explanation is that health professionals might be apprehensive about raising the issue of weight with pregnant women and being perceived as judgmental, especially when the women are overweight (Holton et al., 2017). This idea is interesting as our study showed that the pregnant women considered talking about their gestational weight as positive.

Moreover, our results indicated that very few women who were overweight or obese before pregnancy declared that they had been informed of their weight status, although BMI calculation seems to be a common practice (Kushner and Ryan, 2014). The overweight women in this study, as in the available literature, were nevertheless overexposed to the risk of excessive gestational weight gain (Samura et al., 2016). It has been reported that pre-pregnancy overweight is not systematically diagnosed (Duthie et al., 2013; Nikolopoulos et al., 2017). Stotland et al. (2010) pointed out that health professionals may tend to be more reactive than proactive regarding gestational weight gain problems. Based on the findings in focus groups, they described a common approach to weight gain counseling that could be summarized as “waiting for the cue.” But the cue waited for is mainly a question from the patient. This echoes our observation that the women who felt at ease asking questions more frequently received information. This could contribute to the relative discrepancy between the prevalence of pre-pregnancy overweight and excessive GWG and the proportion of women who actually reported they had been informed about their weight problems.

### An Uninteresting/Useless Subject

Although health professionals believe that gestational weight gain, nutrition, and physical activity are important and are likely to have an impact on the health of women and their babies (Stotland et al., 2010), the literature suggests that physicians may lack the skills, confidence, and resources to change their patients’ behaviors (Stotland et al., 2010; Leiferman et al., 2012; Power and Schulkin, 2017). They may therefore provide information on physical activity less frequently than they should or could.

Studies have reported that health professionals might perceive their advice on weight control as ineffective (Stotland et al., 2010; Power and Schulkin, 2017). For example, they might feel unable to convince pregnant women that it can be dangerous if they gain too much weight or do not maintain a certain level of physical activity, even at the expense of other resources. Our results converge with this observation because the internet/books and family and friends were, respectively, the first and second sources of women’s information about their pregnancy, as reported in the literature (Dalhaug and Haakstad, 2019).

This question of confidence is important since it appears that the most confident physicians are more likely to inform their patients that excessive weight gain increases the risk of pregnancy complications and has possible consequences for their baby (Power and Schulkin, 2017). Also, our results showed that the women who received information about PA more frequently considered it beneficial for their baby’s health and less frequently as dangerous. This suggests that advice has an impact on women’s beliefs and can reassure them about practicing PA during pregnancy. It is important to note that the perception of PA as dangerous and the conviction that it is a source of safety problems have been documented as two barriers to PA in pregnancy (Harrison et al., 2018).

It has also been observed that accessible and consistent information about the positive effects of PA is likely to contribute to adequate PA behaviors during pregnancy (Weir et al., 2010), particularly as the baby’s health is a major motivating factor for lifestyle change (Jelsma et al., 2016).

Health professionals may also think that women do not have much control over their weight and that PA would only put them in a situation of probable failure (Stotland et al., 2010), but our study revealed that only a quarter of the women said they had a hard time following the PA advice they received.

Professionals might also assume that pregnant women are uninterested (Whitaker et al., 2016a) in issues of weight, nutrition and physical activity, and they might anticipate that women will not follow their advice (Leiferman et al., 2012). Such assumptions are important to consider, as more than half the pregnant women in our study reported trying to control their weight, supporting the idea that woman do care about the issue of gestational weight.

Furthermore, our results indicated that although 65% of the pregnant women declared knowing the impacts of PA and nutrition on gestational weight gain, almost three quarters of them reported that they would have liked to know more. It should be noted that in our study, gestational weight gain was considered as an important topic by 65.2% of the women. Our results are in accordance with those of Lindsay et al. (2019) who reported that their weight and weight gain during pregnancy were constantly on their minds for about half of the pregnant women. These statements are probably related to their impact on their health and that of their newborn (Lindsay et al., 2019). In addition, other data in the literature show that gestational weight gain is an important topic for women, in particular by the active search for information on GWG (54.9%) (Willcox et al., 2015), and weighing themselves weekly or fortnightly (57.7%) (Swift et al., 2016).

### A Complex Subject

Health professionals may think they are not sufficiently trained for counseling. They have reported a lack of training in antenatal PA counseling (De Vivo and Mills, 2019; McLellan et al., 2019), and a large majority of those who actually receive a form of training might consider it insufficient (Leiferman et al., 2012).

Our results showed that the women who had the most chance of receiving information were those who felt comfortable asking questions. This indicates that in order to obtain information, women should be able to ask for it; however, they are generally less proactive in seeking advice from health professionals (Walker et al., 2019).

The literature suggests that a non-negligible proportion of health professionals might not be familiar with the PA recommendations in pregnancy (McGee et al., 2018). This is a problem, as a lack of knowledge about the recommendations has been reported as one of the main barriers to PA for women during pregnancy (Connelly et al., 2015).

In addition, some women may experience only limited counseling from midwives because these latter lack sufficient knowledge on this topic (Lindqvist et al., 2018). Thus, the lack of training and knowledge noted above might contribute to the tendency for the women in our study to primarily rely on sources other than health professionals to gather information about their pregnancy. This tendency to seek information elsewhere rather than from medical professionals is understandable in the sense that PA an important subject for pregnant women, as demonstrated in this study and by others (Lindqvist et al., 2018). Yet this could be a cause for concern because other sources might not offer evidence-based advice and could reinforce incorrect beliefs among pregnant women (Shub et al., 2013; Cannon et al., 2019).

### A “Waiting for the Cue” Approach

Special attention to women with weight problems has been signaled in the current recommendations (Institute of Medicine (US) and National Research Council (US) Committee to Reexamine Iom Pregnancy Weight Guidelines, 2009). We thus hypothesized that the higher needs of women with excessive GWG or pre-pregnancy overweight would be associated with an increased likelihood of receiving counseling. However, our results did not indicate this, in line with previous observations (Stengel et al., 2012; Lindsay et al., 2017; Nikolopoulos et al., 2017). The finding that women with special needs do not receive counseling underlines the lack of management of women with weight problems (Kominiarek et al., 2018). However, we observed that the women who felt at ease asking questions during consultations more frequently received information about PA and that women diagnosed by health professionals with excessive GWG were more likely to be talked to about their physical activity. We, as others (Stotland et al., 2012; Arabin et al., 2018), failed to evidence this association in women with a pre-pregnancy diagnosis of overweight. The diagnosis of excessive GWG could be seen as the cue we mentioned above, with health professionals adopting a rather reactive approach regarding weight management in pregnancy. This raises the issue of diagnosing weight problems. We found that the women who were informed of their excessive GWG more frequently received information about PA, but not necessarily the women with a diagnosis of pre-pregnancy overweight/obesity. Yet, this diagnosis, which has an impact on patients’ health behaviors (Banerjee et al., 2016), consequently seems to offer both the opportunity (a cue) to talk about PA in pregnancy and a preliminary step toward counseling. It is therefore concerning to observe that less than a third of the women with pre-pregnancy overweight had been informed of it. We also observed that the diagnosis of excessive gestational weight gain was associated by a fairly high OR (11.38) with receiving PA information. This supports the hypothesis of a “waiting for the cue” approach, with many providers waiting until patients have excessive GWG before addressing the issue and its determinants (Chang et al., 2013).

Finally, although pre-pregnancy overweight diagnosis was not associated with more frequent weight management counseling, it was associated with lower excessive gestational weight gain. This diagnosis thus seems to be associated with different behaviors in pregnant women (May et al., 2013) that the present study was not designed to explore.

### Information/Advice Pregnant Women Would Like to Receive

Talking about weight problems and addressing PA and healthy eating should be included in antenatal care (Jelsma et al., 2016), and women would like to have this information relatively early in pregnancy (Grenier et al., 2020).

Women would like advice and information on the recommendations for PA (Coll et al., 2017), because there is unclear advices (Findley et al., 2020). The health professionals should give counseling for example on type and intensity to practice PA during pregnancy. The experience reported by pregnant women such as “Exercise is necessary for our health,” but “I do not know about the exercises that can be performed during pregnancy” (Fathnezhad-Kazemi and Hajian, 2019) shows a real need for information on their physical activity. Safety discussions at the PA should be approached more often by health professionals. Indeed, pregnant women would like that health professionals lift their fears (Findley et al., 2020), and to know if the practice of physical activity is safe for their health and that of their fetus. Moreover, knowing the benefits in terms of maternal and fetal health is a major motivating factor for practicing PA (Jelsma et al., 2016; Harrison et al., 2018), results also reported in women overweight (Denison et al., 2015; Grenier et al., 2020). Although walking appears to be an easy to implement and often claimed PA for pregnant women (Connolly et al., 2019), health professionals should be discussed appropriate environment to practice. Pregnant women would like to know if there are places to practice for pregnant women, for example “There is no good gym in our neighborhood. I like to go to the gym but I cannot” (Fathnezhad-Kazemi and Hajian, 2019; Grenier et al., 2020).

Regarding weight information, women want to know if they gain too much weight during their pregnancy. In addition, the recommended weight gain based on their initial weight also seems an important topic for them (Stengel et al., 2012). Women desired advice on GWG from their providers specifically tailored for them (Stengel et al., 2012), because the weight − related information in pregnancy are “vague” and “insufficient.” Indeed, Pregnant women would like weight gain goals during pregnancy (Flannery et al., 2020).

Although nutrition appears to be the most frequently provided information in our study, pregnant women generally seem a little disappointed to see that the issue is rarely discussed (Grenier et al., 2020). Generally, women not receiving adequate nutrition education during pregnancy (Lucas et al., 2014). Nutrition advice from a dietician would be appreciated, regarding what (not) to eat and weight management (Jelsma et al., 2016). They want to understand the benefits dietary change during pregnancy (Rundle et al., 2018).

### Limitations

This study has a number of limitations that should be taken into consideration when interpreting and extrapolating the findings. First, the cross-sectional design intrinsically weakens the search for in-depth understanding and causal links. The second important limitation is a non-validated questionnaire, although structured on the basis of the existing literature and pre-tested. It should be recalled that all the data regarding the information received by the women were self-reported. Essentially, this meant that the data reflected the women’s points of view and it cannot be assumed that they reflected the practices of the medical professionals (Duthie et al., 2013; Lozada-Tequeanes et al., 2015). In addition, self-reported data may lead to potential misclassification due to recall biases or social desirability (Sattler et al., 2018).

## Conclusion

Overall, pregnant women receive little counseling on PA and GWG, and this is particularly so for those with weight problems, who are more exposed to excessive GWG. Our results provide additional evidence that the diagnosis of a weight problem impacts behavior and beliefs. Better training for health professionals would increase their knowledge and the frequency of the counseling they give pregnant women to convince them to engage in PA (Malta et al., 2016). It seems an important target and investment for public health policies and policymakers.

## Data Availability Statement

The original contributions presented in the study are included in the article/supplementary material, further inquiries can be directed to the corresponding author.

## Ethics Statement

Ethical review and approval was not required for the study on human participants in accordance with the local legislation and institutional requirements. The study was conducted in accordance with the Declaration of Helsinki and the current local regulations. The patients/participants provided their written informed consent to participate in this study. It was emphasized that participation was voluntary and that they could withdraw from the project at any time with no explanation required.

## Author Contributions

SR and SS designed and set up the study protocol. SR, SS, and SA-J analyzed and interpreted the data and contributed to successive drafts and the revising of the manuscript. SR, SS, OH, EJ, and SA-J read and approved the final manuscript. All authors contributed to the article and approved the submitted version.

## Conflict of Interest

The authors declare that the research was conducted in the absence of any commercial or financial relationships that could be construed as a potential conflict of interest.

## Publisher’s Note

All claims expressed in this article are solely those of the authors and do not necessarily represent those of their affiliated organizations, or those of the publisher, the editors and the reviewers. Any product that may be evaluated in this article, or claim that may be made by its manufacturer, is not guaranteed or endorsed by the publisher.

## Abbreviations

PA: Physical activity
GWG: Gestational weight gain
BMI: Body mass index
ACOG: American College of Obstetricians and Gynecologists.
